# The EKSPECT study: the influence of Expectation modification in Knee arthroplasty on Satisfaction of PatiEnts: study protocol for a randomized Controlled Trial

**DOI:** 10.1186/s13063-018-2821-2

**Published:** 2018-08-14

**Authors:** Jaap J. Tolk, Rob P. A. Janssen, Tsjitske M. Haanstra, Sita M. A. Bierma-Zeinstra, Max Reijman

**Affiliations:** 1 Máxima Medical Center, Department of Orthopedic Surgery and Trauma, PO Box 90052, 5600 PD Eindhoven, The Netherlands; 2 Department of Orthopedic Surgery, Radboud University Medical Center, PO Box 9101, 6500 HB Nijmegen, The Netherlands; 3 Department of General Practice, Erasmus MC, University Medical Center Rotterdam, PO Box 2040, 3000 CA Rotterdam, The Netherlands; 4 Department of Orthopedic Surgery, Erasmus MC, University Medical Center Rotterdam, PO Box 2040, 3000 CA Rotterdam, The Netherlands

**Keywords:** Outcome expectations, Expectation management, Satisfaction, Total knee arthroplasty, Knee osteoarthritis

## Abstract

**Background:**

One out of five patients is unsatisfied to some extent after total knee arthroplasty (TKA). Unmet expectations are the main driver of post-operative dissatisfaction. Improved pre-operative education on realistic expectations for long-term outcome after TKA potentially leads to higher post-operative satisfaction. The effect of expectation modification on post-operative satisfaction in TKA patients has not yet been studied. The primary objective of the presented study is to examine whether an educational module on long-term recovery after TKA will improve patient satisfaction compared to usual pre-operative education.

**Methods:**

The EKSPECT study is a randomized controlled trial. Patients with symptomatic and radiographic knee osteoarthritis who are indicated for a primary TKA will be randomized to the usual pre-operative education (control group) or usual education plus an additional module on realistic expectations for long-term recovery (intervention group). Patients will be naïve to study objective and difference between study groups. Outcome expectations will be measured blinded for group allocation using the HSS Knee Replacement Expectations Survey at baseline (before the intervention), pre-operatively (after the intervention) and fulfillment of expectations at 12-month follow-up. Baseline physical function, quality of life and psychological factors are measured using self-reported questionnaires. The primary outcome measure is satisfaction with treatment result at the 12-month follow-up.

**Discussion:**

The EKSPECT study should provide evidence on the effectiveness of an education module on long-term recovery after TKA to improve treatment satisfaction. If beneficial, the education module is a simple intervention with a low burden for patients, which can easily be implemented in clinical practice.

**Trial registration:**

Dutch Trial Registry registration number: NTR5779. Registered on 17 March 2016.

**Electronic supplementary material:**

The online version of this article (10.1186/s13063-018-2821-2) contains supplementary material, which is available to authorized users.

## Background

Total knee arthroplasty (TKA) is a frequently performed procedure for patients with knee osteoarthritis. The Dutch arthroplasty registry reported an increase from 20,573 TKAs in 2010, to 27,107 in 2016 in the Netherlands [1]. A further increase in these numbers is expected in the future due to aging of the Western population and the growing number of overweight people [1].

TKA is considered an effective intervention for end-stage knee osteoarthritis. Considerable pain reduction, increase in physical function and quality of life can be achieved [2, 3]. The treatment is relatively safe, cost-effective and excellent survival rates are reported, with prosthesis survival of more than 95% at 15 years’ follow-up [2, 4]. Despite these favorable figures, the rate of satisfaction after TKA is consistently reported to be around 80%, leaving one in five patients unsatisfied to some extent after their knee surgery [5, 6].

Patients have multiple expectations regarding the outcome of TKA, mainly concerning relief of pain, improvement in physical functioning and improvement in psychosocial wellbeing [7, 8]. Pre-operative expectations tend to be high, and are often overly optimistic [9, 10]. Frequently, a discrepancy exists between expectations of the patients and those of the surgeon [11], and a substantial number of patients are reported to have unfulfilled expectations after TKA [12]. The fulfillment of pre-operative expectations on treatment outcome is reported as the main determinant of treatment satisfaction after TKA [5, 6, 13, 14]. Patients with unfulfilled expectations, are up to 10 times more likely to be dissatisfied with their treatment results [13, 14]. These findings suggest that more realistic expectations potentially lead to higher post-operative satisfaction.

The current pre-operative patient education is predominantly focused on the process of care and the immediate post-operative period. Previous research has shown that structured pre-operative education on realistic expectations for long-term recovery can alter pre-operative expectations [15]. After such an intervention, expectations of patients are reported to be lower and the rate of discordance between patients’ and surgeons’ expectations is reduced [11, 15]. These findings suggest that specific education about post-operative outcome could lead to more realistic patient expectations. The effect of pre-operative expectation management on post-operative expectation fulfillment, and ultimately better post-operative satisfaction after TKA, has not yet been studied.

## Trial objectives

The primary objective of this randomized controlled trial is to examine whether an additional education module on realistic expectations for long-term recovery of symptoms, physical functioning and psychological issues (intervention group) will improve patient satisfaction after TKA compared to usual pre-operative education (control group).

Furthermore, an analysis will be made to determine if the additional education module on pre-operative expectations of TKA patients leads to change in pre-operative outcome expectations and an increase in post-operative expectation fulfillment. An analysis will be made on the relationship between expectation fulfillment and treatment satisfaction. Additionally, an explorative analysis will be performed on the effect of the additional education module in subgroups of patients, depending on age, gender, severity of symptoms, symptoms of depression, coping mechanisms and degree of pre-operative expectations.

## Methods

### Study design

**Fig. 1:**
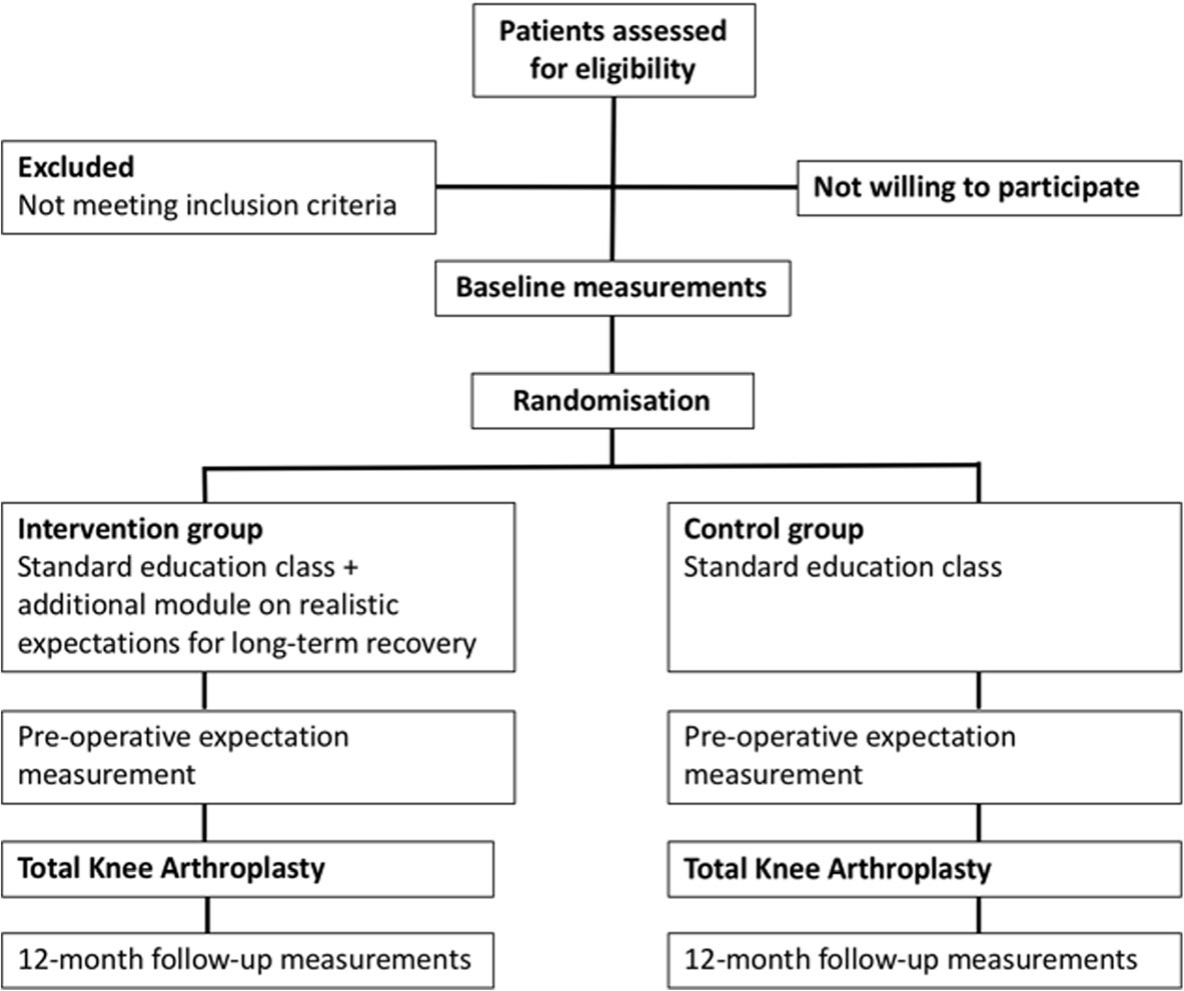
EKSPECT study Consolidated Standards of Reporting Trials (CONSORT)-style flow diagram

The influence of Expectation modification in Knee arthroplasty on Satisfaction of PatiEnts, a randomized Controlled Trial (EKSPECT) study is a randomized, clinical superiority trial, with a parallel-group design and 1:1 allocation ratio. Patients will be randomized to group (a) usual education plus an additional module on realistic expectations for long-term recovery, or in group (b) usual education. Patients will be naïve to study objective and difference between study groups. Measurements will be performed blinded for group allocation at baseline, on day of admission and 12 months after TKA procedure. A flow chart of the study procedures can be found in Fig. 1. The study has been reviewed and approved by the Máxima Medical Center Medical Ethics Committee (registration code NL54671.015.15). The study has been registered in the Dutch Trial Registry (registration number NTR5779) and has not been amended. If protocol amendments are conducted, these will be updated on the Trial Registry record. A Standard Protocol Items: Recommendations for Interventional Trials (SPIRIT) Checklist on the study protocol is provided as Additional file 1.

### Setting

The study will be conducted at the Department of Orthopedic Surgery and Trauma of the Máxima Medical Center. This is a large, non-academic teaching hospital where approximately 350 primary TKAs are performed annually. In total, five experienced orthopedic surgeons perform these procedures.

### Study population

Patients eligible for this trial are patients presenting at the Outpatient Clinic of the Department of Orthopedic Surgery at the Máxima Medical Center, with clinical and radiological knee osteoarthritis, indicated and scheduled for a TKA. The indication for a TKA is set according to the guideline of the Dutch Orthopedic Society [16]. This guideline recommends considering TKA only in patients with radiological knee OA of Kellgren and Lawrence grade ≥ 2 and pain and functional impairment with influence on quality of life, work and/or social life [16].

#### Inclusion criteria

In order to be eligible to participate in this study, a subject must meet the following criterion:

- Symptomatic and radiographic knee osteoarthritis indicated for a primary TKA

#### Exclusion criteria

A potential subject who meets any of the following criteria will be excluded from participation in this study:

- Presence of a medical illness that results in a life expectancy shorter than 1 year
- Presence of TKA of the contralateral side
- Unicompartmental knee arthroplasty
- Staged or bilateral knee arthroplasty
- Insufficient command of the Dutch language
- Legally incompetent adults

The exclusion criteria were chosen because the primary outcome will be measured at the 1-year follow-up and sufficient proficiency of the Dutch language is necessary for understanding of the intervention under study and the completion of patient-reported outcome measures. Furthermore, patients who already have a TKA on the contralateral side will be excluded because their personal experience with knee arthroplasty is known to result in considerable bias on the expectations of outcome after TKA [17]. Patients in whom at baseline it is already evident that they will undergo TKA during the study period (either bilateral or staged) are excluded. These patients are excluded because contralateral knee arthroplasty during the study period is likely to influence satisfaction and functional outcome scores of the index knee as well.

### Recruitment

After being indicated for TKA by their orthopedic surgeon and placed on the waiting list for surgery, eligible patients for the study will be screened by a research nurse. When inclusion and exclusion criteria are met, the patient will be asked to participate in the study. Written information on the study objective and procedures will be provided, and patients will have the possibility to ask additional questions when necessary. The patient will be informed that two education modules are compared in this study, but the difference between the usual care and intervention module is not specified. During this process, patients are blinded for the hypothesis of the study to avoid bias and patient preference for one of the two modules. If a patient is willing to participate, an informed consent form will be signed and baseline measurements registered.

### Randomization

The randomization procedure will be performed after the patient is cleared for surgery in pre-operative screening by the anesthesiologist, and a definitive date for surgery has been determined. Allocation to type of intervention takes place by receiving the consecutive randomization number from the coordinating investigator. A computer-generated randomization list will be used (Research Manager version 5.30.0.6, Cloud9 Software, Deventer, The Netherlands). Patients will be included by each of the five orthopedic surgeons performing and indicating patients for TKA. Because information on outcome after TKA provided by each orthopedic surgeon during the consultation might be different on details, block randomization with variable size of blocks, stratified for orthopedic surgeon is used. This accounts for potential information bias in this regard. All clinical and trial staff will be blinded for allocation during the study procedure. Patients were invited for a specific education class by mail to avoid cross-over, and only the secretary sending the invitation letters for the education sessions is unblinded. Attendance of the education class in the intervention and control arms is recorded blinded using the data management software. Data analysis will be obtained and analyzed blinded for group allocation. Only after data analysis will unblinding take place.

### Intervention

#### Theoretical framework / rationale for using an education module to increase post-operative satisfaction

Patient expectations have been defined as “anticipations that given events are likely to occur during or as a result of medical care,” and expectations regarding treatment result are defined as “outcome expectations” [18–21]. The present study addresses probabilistic outcome expectations; what does the patient think is the most likely long-term result after TKA? [18–20].

Different hypotheses on the effect of expectation modification on treatment outcome and satisfaction have been suggested. In general, positive expectation of recovery has shown to be associated with better health outcomes [22, 23] and lower recovery expectations increased the risk of persistent activity limitations [24]. The strength of the relation depends on the clinical conditions and the method of expectation measurement used [22]. Expectations that are too low or negative can result in less motivation to obtain full benefit from the surgery, and increasing patient expectations is suggested to improve treatment outcome [25, 26]. Suggested explanations for this positive effect are that higher expectations result in anxiety reduction, better cooperation with treatment and beneficial coping mechanisms [27, 28]. There is evidence that expectations are a mechanism by which placebos have their effects [25]. Utilization of this placebo effect as an intervention to obtain positive health-related effects has been shown in laboratory settings with predominantly healthy volunteers, [28] but these positive results have not been consistently reproduced in clinical research [29]. To the authors’ knowledge there are no intervention studies available on the effect of increasing expectations to improve treatment outcome in TKA patients. So, it remains to be seen if this potential positive effect actually occurs and to what extent outcome improvement can be obtained.

On the other hand, unrealistically high expectations can result in discouraged patients and non-adherence with recommendations post-operatively. Total knee arthroplasty patients often have overly optimistic pre-operative expectations when compared to surgeons [11] and when compared to actual outcome [9, 30]. Furthermore unmet pre-operative expectations are strongly related to post-operative dissatisfaction [5, 6]. These findings are in line with the expectancy-disconfirmation theory, which states that satisfaction is a function of expectations, perceived performance and disconfirmation of beliefs [31]. Therefore, in the authors’ opinion an education module should not result in overly optimistic expectations, as these pose the risk of expectation disconfirmation with subsequent patient dissatisfaction.

In conclusion, expectation management in knee arthroplasty patients is thought to have an effect on post-operative satisfaction and outcome through various pathways. Whereas, on the one hand, expectations should be high enough to fully benefit from the placebo effect, on the other hand, unmet expectations can result in higher dissatisfaction rates after treatment. “Optimistic realism” is suggested as the appropriate balance between preventing dissatisfaction and optimizing context effects [32]. In the authors’ view both aspects are important, and it is key to identify the patients with unrealistic expectations; either too high or too low and adjust these accordingly. Therefore, the aim of the proposed additional education module is to achieve realistic expectations on long-term recovery after TKA.

#### Intervention arm

The intervention under study is a joint-specific educational module on long-term recovery after TKA (12 months post-operative). The additional education module is an extension to the pre-operative education program as described for the control group. Information in the education module is based on an academic literature study, the expert opinion of TKA surgeons in our own clinic and a survey among members of the Dutch Knee Arthroplasty Society [33]. The final module was written and approved by an expert panel consisting of an experienced knee surgeon (RJ), an orthopedic surgery resident and PhD student (JT) and a researcher specialized in osteoarthritis treatment outcome (MR).

Previous research has identified a set of important expectations that are often not fulfilled in TKA patients [7, 8, 30]. These items are incorporated in the Hospital for Special Surgery (HSS) Knee Replacement Expectations Survey [7, 34]. Items addressed in this survey were used as the framework for the education module. The module describes what patients can generally expect 12 months post surgery concerning the amount of pain, functioning in daily life (e.g., walking, rising from a chair, stair climbing); performing social activities (e.g., hobbies, sport activities); and psychological wellbeing (e.g., psychological wellbeing, interactions with others). Information is provided for the most likely outcome for the whole population of TKA patients. Additionally, modifying factors are addressed that predict higher or lower outcome for an individual patient; age, medical co-morbidity, Body Mass Index (BMI), psychosocial factors, pain severity and pre-operative functional status [35, 36]. The module consists of a group-based lecture of approximately 15 min, given by the senior author (JT, orthopedic surgery resident and PhD student), and information on realistic outcome expectations after TKA in writing (Additional file 2). When patients have additional questions or concerns regarding realistic expectations on treatment outcome, these will be addressed after the lecture. The module was formatted into the existing standard program with a session once a month for approximately five to 10 patients, and is in concordance with recommendations on education class structure [37].

In summary, the education module states the following: overall good effect for long-term recovery after TKA can be expected [33]. Most improvement can be expected for the items pain relief, ability to perform daily activities and walking short to medium distances [10, 33, 38]. Significant pain relief is achieved in most patients after TKA, nevertheless, some residual pain is common [10, 38]. Impairment in daily activities is likely to decrease. At 12 months much better or better activity of daily living function is reported by more than 90% of patients, only 4–8% of patients report some problems in daily living [10, 39]. A large improvement or return to normal can be expected on walking short and medium distances [10, 33, 40]. For longer distances, some limitations are likely to remain [10, 33]. Least improvement can be expected for the activities of kneeling, squatting, stair negotiation and the ability to exercise or participate in sports [33, 38]. Approximately 80% of patients report to be unable to kneel or squat without knee symptoms after TKA [41, 42]. The rate of return to sport is dependent on patient characteristics and type of sports. The intensity of activity to which patients return tends to be less than before surgery; 94% are able to do low-impact sports but only 43% are reported to return to high-impact sports [43, 44].

The above summary is not exhaustive, but in the actual education module realistic expectations for all items of the HSS Knee Replacement Survey are addressed. Improvement can be expected in the domains of pain, function, activities and psychological wellbeing but return to normal is not likely to occur. Limitations can predominantly be expected in more demanding physical activities and sports.

#### Control arm

The standard pre-operative education program consists of information about the perioperative period, but does not include information on long-term recovery. The admission process, details about the anesthesia process, surgical technique, complications, pain management, the direct post-operative recovery and rehabilitation during the first 6 weeks post-operative are addressed. During a 120-min multidisciplinary pre-operative class an anesthesiology assistant, physiotherapist, orthopedic nurse and orthopedic surgeon teach a 30-min module each. The information is summarized in a brochure for the patients. This education module currently is the standard care in Máxima Medical Center for all TKA patients.

### Outcome measurement

**Fig. 2:**
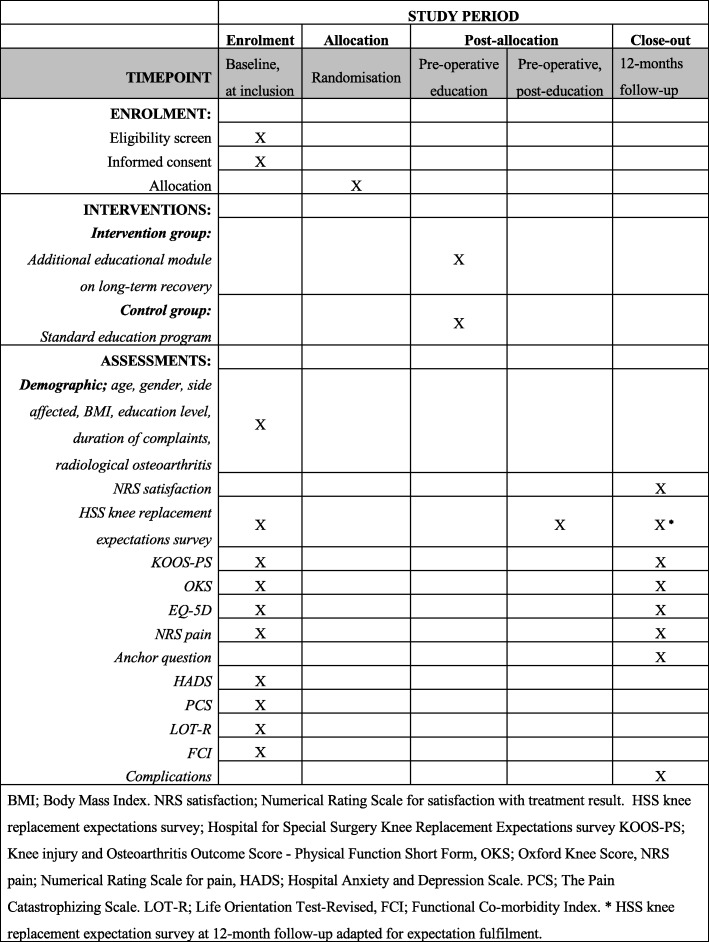
Standard Protocol Items: Recommendation for Interventional Trials (SPIRIT) summary of study timing and activities

Outcome measures used at the different time points can be found in Fig. 2.

#### Primary outcome measure

The primary outcome measure is a Numeric Rating Scale (NRS) score for satisfaction with treatment result at 12 months’ follow-up. The NRS satisfaction score is a self-reported measure for patient satisfaction [45]. Patients are asked to answer the question: “How satisfied are you (in general) about the result of your knee operation?” on a scale ranging from 0 (very dissatisfied) to 10 (very satisfied) [45]. “Very satisfied with treatment result” is defined as an NRS satisfaction score of ≥ 8.

#### Patient characteristics

At baseline, patient characteristics will be obtained: age, gender, side affected, height, weight, education level, duration of complaints and radiological osteoarthritis severity scored according to the Kellgren and Lawrence grading system [46].

#### Secondary outcome measures

##### Hospital for Special Surgery (HSS) Knee Replacement Expectations Survey

Patient expectations will be evaluated by the Dutch version of the HSS Knee Replacement Survey [34]. Measurements will be obtained at baseline (after inclusion at the outpatient clinic, before pre-operative education), at admission for surgery (after the pre-operative education) and fulfillment of expectations 12 months post-operatively. The HSS Knee Replacement Expectations Survey is a 19-item, self-administered survey, measuring probability-based outcome expectations in the domains of pain, function, activities and psychological wellbeing [7, 15]. The survey is reliable, validated, and considered to be a high-quality expectation assessment instrument [7, 34, 47]. Patients will be asked how much improvement they expect for each item; the following response format will be used: “complete improvement or back to normal,” “a lot of improvement,” “a moderate amount of improvement,” “a little improvement” or “this expectation does not apply to me/I do not have this expectation” [15, 34]. The total score ranges from 0 to 76, which will be recoded on a 100-point scale, with a higher score representing higher expectations [15, 34].

To assess to what extent expectations have been fulfilled 12 months post-operatively, the expectation fulfillment version of the HSS Knee Replacement Expectations Survey will be used [12]. Unfortunately no evaluation of the measurement properties of this modification are available. The perceived actual outcome is scored with the same answer options and score calculation as used for pre-operative measurement [12].

##### Knee injury and Osteoarthritis Outcome Score – Physical Function Short Form (KOOS*-*PS)

The KOOS-PS Dutch version is a seven-item knee-specific questionnaire for measurement of the construct physical function. From 5-point Likert scale questions, a normalized score is calculated ranging from no difficulty (0) to extreme difficulty (100) [48]. The KOOS-PS has good reliability, validity and ability to detect change over time in knee osteoarthritis patients [48, 49].

##### Oxford Knee Score (OKS)

The Dutch version of the OKS is a 12-item patient-reported measure for assessment of pain and function after TKA. Each question consists of a 5-point Likert scale, leading to a total score ranging from a best functional score of 12 to the worst functional outcome score of 60 [50]. It is short, reproducible, valid and sensitive to clinically important changes [49, 50].

##### EQ-5D

The Dutch version of the EuroQol 5D-3 L (EQ-5D) is a self-reported questionnaire, measuring generic health status [51]. The EQ-5D comprises five questions scored on a 3-point Likert scale and a Visual Analogue Scale (EQ VAS) where the endpoints are labeled “Best imaginable health state” and “Worst imaginable health state.” From the five questions a sum score can be calculated: 1 represents the best possible health state and lower scores imply a lower health state [51]. The EQ-5D has good reliability and validity in knee osteoarthritis patients [52].

##### NRS pain

The NRS score for pain during activity and at rest (NRS pain) during the past week will be measured on an 11-point scale. A score of 0 represents “no pain” and a score of 10 represents “worst imaginable pain.” The NRS has good reliability and responsiveness [53].

##### Anchor question

At the follow-up 12 months post-operative a 7-point Likert scale anchor question will be scored for change in activities of daily living. The question “How has your general daily functioning changed since the operation on your knee?” can be responded to on a 7-point scale ranging from 1 (a lot worse) to 7 (very much improved).

##### Hospital Anxiety and Depression Scale (HADS)

The Dutch version of the HADS is a 14-item questionnaire for the measurement of anxiety and depressive symptoms [54, 55]. Seven items that relate to anxiety and seven items that relate to depression are rated on a 4-point scale. For both subscales, a sum score will be calculated ranging from 0 meaning no symptoms to 21 meaning severe symptoms.

##### The Pain Catastrophizing Scale (PCS)

The Dutch version of the PCS is a reliable and valid self-reported measure of catastrophizing [56]. Catastrophizing is defined as an exaggerated negative orientation toward noxious stimuli and is an important aspect of pain experience and coping [56]. The PCS consists of 13 5-point scale questions about thoughts and feelings on pain experience. Subscales for rumination, magnification and helplessness have been defined. A total score can be calculated ranging from 0 (no catastrophizing) to 52 (extreme catastrophizing).

##### Life Orientation Test-Revised (LOT-R)

The Dutch version of the LOT-R assesses the constructs dispositional optimism and pessimism and has satisfactory psychometric properties [57, 58]. This self-reported measure consists of 10 items. Three items (1, 4 and 10) assess optimism, three items (3, 7 and 9) assess pessimism, and four are filler items. Response categories range from “strongly agree” to “strongly disagree” on a 5-point Likert scale. Scores for the two subscales can be calculated and the total score is calculated by adding the optimism and the inverted pessimism score.

##### Functional Co-morbidity Index (FCI)

Co-morbidity is scored using the FCI. The index consists of a list of 18 diagnoses associated with declining function [59]. One point is assigned to each diagnosis, and the points are summarized, giving the patient a score of between 0 and 18 [59]. The FCI is considered a reliable and valid tool for assessment of co-morbidity in osteoarthritis patients [60].

##### Complications

At 1-year follow-up complications that have occurred will be scored as advised by the Knee Society [61]. This classification system allows structured reporting of occurrence and severity of 22 potential complications after TKA [61]. Adverse events are deemed unlikely due to the study design, occurrence will be monitored and reported.

### Sample size and power calculations

The calculation of the number of patients needed for this trial was based on the following assumptions. Specific pre-operative information can lead to more realistic patient expectations, which subsequently leads to a higher probability of fulfilled expectations [15]. Furthermore, patients who have fulfilled expectations are more often satisfied with the results of TKA [13, 14]. Therefore, we hypothesize that patients are more often (very) satisfied with treatment outcome 1 year post-operative. Based on more realistic expectations after improved pre-operative education on long-term recovery, previous work has shown that 50% of patients are very satisfied after TKA, 23% somewhat satisfied, 11% neutral, 9% somewhat dissatisfied, and 7% were very dissatisfied with the outcome [62]. In our calculation, we used a power of 80% (type II error of 20%) and an alpha of 0.05 (type I error). To increase the proportion of very satisfied patients in the knee population with 20% (from 50% of very satisfied patients to 70% very satisfied patients), 90 patients are required in each group (180 TKA patients in total). The final total sample size required is 204 knee patients, to accommodate a 15% potential dropout rate.

### Data management

All data will be handled confidentially and anonymized in compliance with the Dutch Personal Data Protection Act (“Wet Bescherming Persoonsgegevens”). Questionnaires are collected digitally and the patient study data will be stored coded using data management software (Research Manager version 5.30.0.6, Cloud9 Software, Deventer, The Netherlands). Each patient receives an anonymized study number that is used for all documentation, study reports and publications. The key of this study number will be handled by an independent researcher. All data will be stored during the study period, and when the study is finished, the research files are stored for 15 years in the Máxima Medical Center research archive. The Medical Research Ethics Committee deemed the study as a “low-risk” study. Therefore, no Data Monitoring Committee was recommended at the time of ethical approval. The Medical Research Ethics Committee will be informed yearly on the inclusion rate, adverse events and study results.

### Statistical analysis

The primary analysis will be performed according to intention-to-treat principles following the original group allocation regardless of the intervention that they actually received. Missing data will be accounted for by a multiple imputation technique. A secondary per-protocol analysis will be performed, including only those patients who completed the treatment originally allocated. Reasons for cross-over will be explored. Distribution of all variables will be tested by the Shapiro-Wilk test. For the normally distributed variables, parametric tests will be used. For the variables that were not normally distributed non-parametric tests will be used.

The primary outcome will be analyzed by using logistic regression analyses (patient satisfaction with the 12 months’ results of TKA as dependent and intervention as independent variable), variables of which a priori are known to be associated with patient satisfaction, based on previous studies or based on a strong clinical rationale, will be considered as covariates in the primary analysis. These covariates are age, gender, BMI and baseline expectations as assessed by the HSS Knee Replacement Survey, anxiety, depression. The assumptions of constant variance and linear relationships will be assessed using scatter plots. Should any of these assumptions seriously fail, then transformation of the dependent or independent variable(s) (where applicable) will be used. The choice of which transformation (e.g., square root, logarithm) will be used based on the specific distribution of the residuals. Similar analyses will be performed for the secondary outcome parameters. Change in outcome expectations before and after pre-operative education (baseline vs pre-operative), and difference between intervention and control group in change in baseline and pre-operative expectations will be analyzed using linear regression analyses. Statistical calculations will be made using IBM® SPPS software, version 19.0.

## Discussion

The EKSPECT study is the first trial to analyze the potential of expectation management to increase post-operative satisfaction in TKA patients. Meeting pre-operative expectations is known to be of major influence on post-operative satisfaction after TKA. More realistic expectations will potentially lead to higher post-operative satisfaction. The present study will analyze the effect of improved pre-operative education, with specific attention to realistic expectations for long-term functional recovery after TKA, on post-operative satisfaction.

Strengths of the study include the sound methodological framework, with double-blind, randomized allocation and assessment of the intervention. Secondly, in previous research on expectation management, poor measurement methods and inconsistent definition of constructs under study are common [18, 19]. It is, therefore, recommended to clearly define the construct measured and the theoretical framework it derives from, to allow accurate interpretation of the results [19]. In the authors’ opinion these factors are adequately addressed in the present study design. Thirdly, the study procedures will be fully integrated into the current clinical process. This aids in the generalizability of the study results, limits the burden for the study population and increases the likelihood for patients to be willing to participate.

A limitation of the proposed study is that the content of the intervention education module is based on what patients undergoing TKA consider the most important expectations [7, 8, 30], and patient experiences after TKA as previously reported in the academic literature. Although a patient-centered approach was used in the design, patients were not directly involved in the construction of the module.

Furthermore, a limitation of the proposed study could be that the intervention provides education on realistic expectations for the general TKA population. Potential individual modifiers influencing outcome after TKA are provided, but the prediction is not individualized. Currently available prediction models do not seem suitable for individualized expectation management [63]. The existing outcome prediction tools mainly focus on identifying patients most likely not to benefit from TKA, but do not provide specific information on pain and functional outcome to guide pre-operative expectation management [63]. Furthermore, such an individual approach would increase the burden for patients and medical staff to a much larger extent than the proposed additional education module. Therefore, this pragmatic design was chosen to keep the burden for the patient as low as possible and increase the possibility of future implementation when positive results are found.

For the study, osteoarthritis patients with an indication for primary TKA will included in a large, non-academic hospital in the Netherlands. It is known that patients from different countries have different expectations regarding TKA [64]. Expectations and satisfaction rates are also known to differ across indications for TKA [6]. These factors might limit the generalizability of the study results to some extent.

### Trial status

The study inclusion has started in the summer of 2016. Final results are anticipated mid-2019, and the authors aim to report on the findings shortly thereafter.

## Additional files

Additional file 1: SPIRIT 2013 Checklist. (DOC 122 kb)

Additional file 2: Leaflet additional educational module on long-term recovery after TKA. (PDF 701 kb)

## Abbreviations

BMI: Body Mass Index
CONSORT: Consolidated Standards of Reporting Trials
EKSPECT: The influence of Expectation modification in Knee arthroplasty on Satisfaction of PatiEnts, a randomized Controlled Trial
FCI: Functional Co-morbidity Index
HADS: Hospital Anxiety and Depression Scale
HSS Knee Replacement Expectations Survey: Hospital for Special Surgery Knee Replacement Expectations Survey
KOOS-PS: Knee injury and Osteoarthritis Outcome Score–Physical Function Short Form
LOT-R: Life Orientation Test-Revised
NRS: Numeric Rating Scale
OKS: Oxford Knee Score
PCS: Pain Catastrophizing Scale
SPIRIT: Standard Protocol Items: Recommendation for Interventional Trials
TKA: Total knee arthroplasty

## References

[1] Dutch Arthroplasty Register (LROI). Online LROI annual report 2017: 10 years of registration, a wealth of information. ‘s-Hertogenbosch: LROI organization; 2017.

[2] Ruiz D, Koenig L, Dall TM, Gallo P, Narzikul A, Parvizi J (2013). The direct and indirect costs to society of treatment for end-stage knee osteoarthritis. J Bone Jt Surg Am.

[3] Tolk JJ, Janssen RPA, Prinsen CAC, Latijnhouwers DAJM, van der Steen MC, Bierma-Zeinstra SMA, et al. The OARSI core set of performance-based measures for knee osteoarthritis is reliable but not valid and responsive. Knee Surg Sport. Traumatol. Arthrosc. 2017; epub ahead:of print 10.1007/s00167-017-4789-y 29128879

[4] McCalden RW, Hart GP, MacDonald SJ, Naudie DD, Howard JH, Bourne RB (2017). Clinical results and survivorship of the GENESIS II total knee arthroplasty at a minimum of 15 years. J Arthroplasty.

[5] Noble PC, Conditt MA, Cook KF, Mathis KB (2006). The John Insall award: patient expectations affect satisfaction with total knee arthroplasty. Clin. Orthop. Relat. Res.

[6] Dunbar MJ, Richardson G, Robertsson O (2013). I can’t get no satisfaction after my total knee replacement: rhymes and reasons. Bone Joint J.

[7] Mancuso CA, Sculco TP, Wickiewicz TL, Jones EC, Robbins L, Warren RF. Patients’ expectations of knee surgery. J Bone Jt. Surg Am. 2001;83–A:1005–12. 10.2106/00004623-200107000-00005 11451969

[8] Smith EJ, Soon V-L, Boyd A, McAllister J, Deakin A, Sarungi M (2016). What do Scottish patients expect of their total knee arthroplasty?. J. Arthroplasty.

[9] Mannion AF, Kämpfen S, Munzinger U, Kramers-de QI (2009). The role of patient expectations in predicting outcome after total knee arthroplasty. Arthritis Res Ther.

[10] Nilsdotter AK, Toksvig-Larsen S, Roos EM (2009). Knee arthroplasty: are patients’ expectations fulfilled? A prospective study of pain and function in 102 patients with 5-year follow-up. Acta Orthop.

[11] Ghomrawi HMK, Mancuso CA, Westrich GH, Marx RG, Mushlin AI (2013). Discordance in TKA expectations between patients and surgeons knee. Clin. Orthop. Relat. Res.

[12] Tilbury C, Haanstra TM, Leichtenberg CS, Verdegaal SHM, Ostelo RW, de Vet HCW (2015). Unfulfilled expectations after total hip and knee arthroplasty surgery: there is a need for better preoperative patient information and education. J. Arthroplasty.

[13] Bourne RB, Chesworth BM, Davis AM, Mahomed NN, Charron KDJ (2010). Patient satisfaction after total knee arthroplasty: who is satisfied and who is not?. Clin Orthop Relat Res.

[14] Hamilton DF, Lane JV, Gaston P, Patton JT, Macdonald D, Simpson AH, Howie CR (2013). What determines patient satisfaction with surgery? A prospective cohort study of 4709 patients following total joint replacement. BMJ Open.

[15] Mancuso C, Graziano S, Briskie L, Peterson M, Pellicci P, Salvati E (2008). Randomized trials to modify patients’ preoperative expectations of hip and knee arthroplasties. Clin Orthop Relat Res.

[16] Dutch Orthopaedic Association. Guideline Total Knee Arthroplasty. NOV, 's Hertogenbosch; 2014.

[17] Hepinstall MS, Rutledge JR, Bornstein LJ, Mazumdar M, Westrich GH (2011). Factors that impact expectations before total knee arthroplasty. J. Arthroplasty.

[18] Haanstra TM, van den Berg T, Ostelo RW, Poolman RW, Jansma EP, Jansma IP (2012). Systematic review: do patient expectations influence treatment outcomes in total knee and total hip arthroplasty?. Health Qual Life Outcomes.

[19] Tolk JJ, Haanstra TM, Reijman M (2016). Letter to the editor on “What do Scottish patients expect of their total knee arthroplasty?”. J Arthroplasty.

[20] Kravitz RL (1996). Patients’ expectations for medical care: an expanded formulation based on review of the literature. Med Care Res Rev.

[21] Uhlmann RF, Inui TS, Carter WB. Patient requests and expectations. Definitions and clinical applications. Med Care. 1984;22:681–5. 10.1097/00005650-198407000-00011 6748787

[22] Mondloch MV, Cole DC, Frank JW (2001). Does how you do depend on how you think you’ll do? A systematic review of the evidence for a relation between patients’ recovery expectations and health outcomes. Can Med Assoc J.

[23] Jain D, Nguyen L-CL, Bendich I, Nguyen LL, Lewis CG, Huddleston JI (2017). Higher patient expectations predict higher patient-reported outcomes, but not satisfaction, in total knee arthroplasty patients: a prospective multicenter study. J Arthroplasty.

[24] Iles RA, Davidson M, Taylor NF, O’Halloran P (2009). Systematic review of the ability of recovery expectations to predict outcomes in non-chronic non-specific low back pain. J Occup Rehabil.

[25] Crow R, Gage H, Hampson S, Hart J, Kimber A, Thomas H (1999). The role of expectancies in the placebo effect and their use in the delivery of health care: a systematic review. Health Technol Assess.

[26] Ebrahim S, Malachowski C, Kamal El Din M, Mulla SM, Montoya L, Bance S (2014). Measures of patients’ expectations about recovery: a systematic review. J Occup Rehabil.

[27] Flood AB, Lorence DP, Ding J, McPherson K, Black NA (1993). The role of expectations in patients’ reports of post-operative outcomes and improvement following therapy. Med Care.

[28] Colloca L, Miller FG (2011). Harnessing the placebo effect: the need for translational research. Philos. Trans R Soc Lond B Biol Sci.

[29] Di Blasi Z, Harkness E, Ernst E, Georgiou A, Kleijnen J (2001). Influence of context effects on health outcomes: a systematic review. Lancet.

[30] Tilbury C, Haanstra TM, Leichtenberg CS, Verdegaal SHM, Ostelo RW, de Vet HCW (2016). Unfulfilled expectations after total hip and knee arthroplasty surgery: there is a need for better preoperative patient information and education. J. Arthroplasty.

[31] Oliver RL (1977). Effect of expectation and disconfirmation on postexposure product evaluations: an alternative interpretation. J Appl Psychol.

[32] Haanstra TM. Patients’ expectations. Determinants, mechanisms and impact on clinical outcomes. (PhD Thesis). VU medical center; 2015.

[33] Tolk JJ, van der Steen M, Janssen RPA, Reijman M (2017). Total knee arthroplasty: what to expect ? A survey of the members of the Dutch Knee Society on long- term recovery after total knee arthroplasty. J Knee Surg.

[34] van den Akker-Scheek I, van Raay JJ, Reininga IH, Bulstra SK, Zijlstra W, Stevens M (2010). Reliability and concurrent validity of the Dutch hip and knee replacement expectations surveys. BMC Musculoskelet. Disord.

[35] Lingard EABMM, Katz JNMM, Wright EAP, Sledge CBM (2004). The Kinemax Outcomes Group. Predicting the outcome of total knee arthroplasty. J. Bone Jt. Surg Am.

[36] Singh JA, O’Byrne M, Harmsen S, Lewallen D (2010). Predictors of moderate-severe functional limitation after primary total knee arthroplasty (TKA): 4701 TKAs at 2-years and 2935 TKAs at 5-years. Osteoarthr Cartil.

[37] Edwards PK, Mears SC, Lowry BC (2017). Preoperative education for hip and knee replacement: never stop learning. Curr. Rev. Musculoskelet. Med.

[38] Husain A, Lee G (2015). Establishing realistic patient expectations following total knee arthroplasty. J Am Acad Orthop Surg.

[39] Dahm DL, Barnes SA, Harrington JR, Sayeed SA, Berry DJ (2008). Patient-reported activity level after total knee arthroplasty. J Arthroplast.

[40] Abbasi-Bafghi H, Fallah-Yakhdani HR, Meijer OG, de Vet HC, Bruijn SM, Yang L-Y (2012). The effects of knee arthroplasty on walking speed: a meta-analysis. BMC Musculoskelet Disord.

[41] Weiss JM, Noble PC, Conditt MA, Kohl HW, Roberts S, Cook KF, et al. What functional activities are important to patients with knee replacements? Clin Orthop Relat Res. 2002:172–88. 10.1097/00003086-200211000-00030 12439258

[42] Hepinstall MS, Ranawat AS, Ranawat CS (2010). High-flexion total knee replacement: functional outcome at one year. HSS J.

[43] Jassim SS, Douglas SL, Haddad FS (2014). Athletic activity after lower limb arthroplasty: a systematic review of current evidence. Bone Joint J.

[44] Witjes S, Gouttebarge V, Kuijer PPFM, van Geenen RCI, Poolman RW, Kerkhoffs GMMJ (2016). Return to sports and physical activity after total and unicondylar knee arthroplasty: a systematic review and meta-analysis. Sport Med.

[45] Kahlenberg CA, Nwachukwu BU, McLawhorn AS, Cross MB, Cornell CN, Padgett DE. Patient satisfaction after total knee replacement: a systematic review. HSS J. 2018:192–201. 10.1007/s11420-018-9614-8 PMC6031540 29983663

[46] Kellgren JH, Lawrence JS (1957). Radiological assessment of osteo-arthrosis. Ann Rheum Dis.

[47] Zywiel MG, Mahomed A, Gandhi R, Perruccio AV, Mahomed NN (2013). Measuring expectations in orthopaedic surgery: a systematic review. Clin. Orthop. Relat. Res.

[48] de Groot IB, Favejee MM, Reijman M, Verhaar JA, Terwee CB (2008). The Dutch version of the Knee Injury and Osteoarthritis Outcome Score: a validation study. Health Qual Life Outcomes.

[49] Collins NJ, Misra D, Felson DT, Crossley KM, Roos EM (2011). Measures of knee function: International Knee Documentation Committee (IKDC) Subjective Knee Evaluation Form, Knee Injury and Osteoarthritis Outcome Score (KOOS), Knee Injury and Osteoarthritis Outcome Score Physical Function Short Form (KOOS-PS), Outcome. Arthritis Care Res. (Hoboken).

[50] Haverkamp D, Breugem SJM, Sierevelt IN, Blankevoort L, van Dijk CN (2005). Translation and validation of the Dutch version of the Oxford 12-item Knee Questionnaire for knee arthroplasty. Acta Orthop.

[51] Rabin R, de Charro F (2001). EQ-5D: a measure of health status from the EuroQol Group. Ann Med.

[52] Conner-Spady BL, Marshall DA, Bohm E, Dunbar MJ, Loucks L, Al Khudairy A (2015). Reliability and validity of the EQ-5D-5L compared to the EQ-5D-3L in patients with osteoarthritis referred for hip and knee replacement. Qual. Life Res.

[53] Ruyssen-Witrand A, Fernandez-Lopez CJ, Gossec L, Anract P, Courpied JP, Dougados M (2011). Psychometric properties of the OARSI/OMERACT osteoarthritis pain and functional impairment scales: ICOAP, KOOS-PS and HOOS-PS. Clin Exp Rheumatol.

[54] Bjelland I, Dahl AA, Haug TT, Neckelmann D (2002). The validity of the Hospital Anxiety and Depression Scale. An updated literature review. J Psychosom Res.

[55] Spinhoven P, Ormel J (1997). A validation study of the Hospital Anxiety and Depression Scale (HADS) in different groups of Dutch subjects. Psychol Med.

[56] Sullivan MJL, Bishop SR, Pivik J (1995). The Pain Catastrophizing Scale: development and validation. Psychol Assess.

[57] ten Klooster PM, Weekers AM, Eggelmeijer F, van Woerkom JM, Drossaert CHC, Taal E (2010). Optimisme en / of pessimisme: factorstructuur van de Nederlandse Life Orientation Test Revised. Psychol Gezondh.

[58] Glaesmer H, Rief W, Martin A, Mewes R, Brähler E, Zenger M (2012). Psychometric properties and population-based norms of the Life Orientation Test Revised (LOT-R). Br J Health Psychol.

[59] Groll DL, To T, Bombardier C, Wright JG (2005). The development of a comorbidity index with physical function as the outcome. J Clin Epidemiol.

[60] Bjorgul K, Novicoff WM, Saleh KJ (2010). Evaluating comorbidities in total hip and knee arthroplasty: available instruments. J Orthop Traumatol.

[61] Iorio R, Della Valle CJ, Healy WL, Berend KR, Cushner FD, Dalury DF (2014). Stratification of standardized TKA complications and adverse events: a brief communication. Clin Orthop Relat Res.

[62] Vissers MM, De Groot IB, Reijman M, Bussmann JB, Stam HJ, Verhaar JAN. Functional capacity and actual daily activity do not contribute to patient satisfaction after total knee arthroplasty. BMC Musculoskelet Disord. 2010;11 10.1186/1471-2474-11-121 PMC2896921 20553584

[63] Riddle DL, Golladay GJ, Jiranek WA, Perera RA (2017). External validation of a prognostic model for predicting nonresponse following knee arthroplasty. J. Arthroplasty.

[64] Lingard EA, Sledge CB, Learmonth ID (2006). Patient expectations regarding total knee arthroplasty: differences among the United States, United kingdom, and Australia. J. Bone Jt. Surgery, Am.

