# Supplementary material for: The EKSPECT study: the influence of Expectation modification in Knee arthroplasty on Satisfaction of PatiEnts: study protocol for a randomized Controlled Trial
Source: Trials. 2018 Aug 14;19:437. doi: 10.1186/s13063-018-2821-2 (PMC6092877; doi:10.1186/s13063-018-2821-2)
Supplement: Supplementary file 2 — Leaflet additional educational module on long-term recovery after TKA. (PDF 701 kb) [file 13063_2018_2821_MOESM2_ESM.pdf]

Psychological well-being

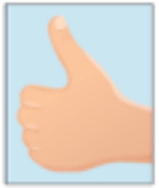

Patients often feel better after knee replacement. If there was a question of gloom, there is often a noticeable improvement after the operation. On average, psychological well-being increases slightly after a total knee prosthesis.

Sports and recreation

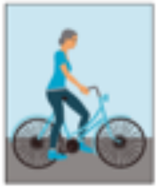

You can expect a moderate improvement for these activities. Sports such as cycling, hiking and golf generally are without problems. More intensive sports in which the knee prosthesis is heavily loaded, such as football, running, indoor sports, hockey and contact sports are not recommended.

Orthopedisch Centrum Máxima
(040) 888 86 00
ocm@mmc.nl
https://ocm.mmc.nl

Locatie Eindhoven:
Ds. Th. Fliednerstraat 1
Postbus 90 052
5600 PD Eindhoven

Locatie Veldhoven:
de Run 4600
Postbus 7777
5500 MB Veldhoven

www.mmc.nl

De informatie in deze folder is van algemene aard en is bedoeld om u een beeld te geven van de zorg en voorlichting die u kunt verwachten. In uw situatie kunnen andere adviezen of procedures van toepassing zijn. Deze folder is dan ook slechts een aanvulling op de specifieke (mondelinge) voorlichting van uw specialist of behandelaar.

MMC 220.233_04-16

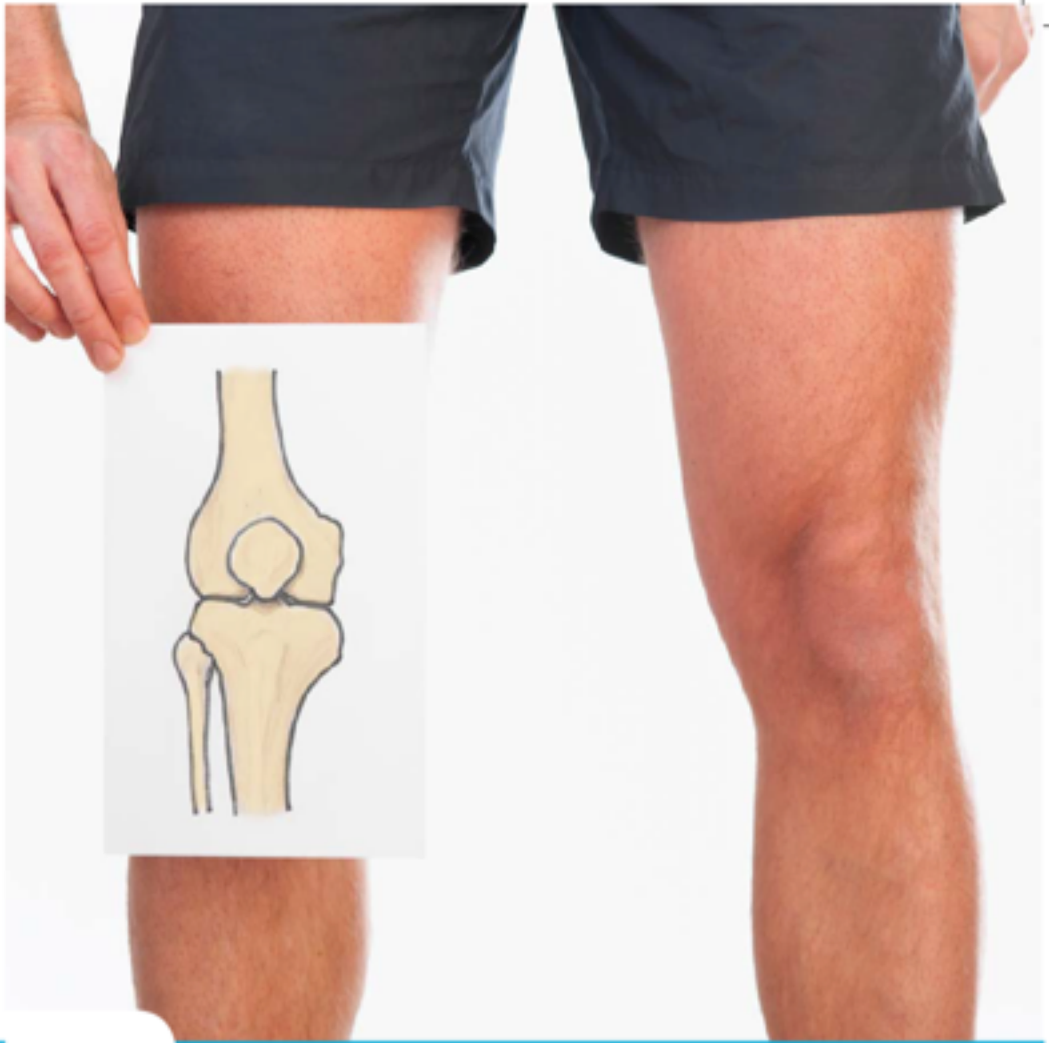

Long term recovery after total knee arthroplasty

Orthopedic Centre Máxima

What to expect from your knee replacement in the long term?

In this leaflet you will find information about what you can expect 1 year after a total knee prosthesis. The results shown are an average. Your individual outcome may deviate from this, partly because of your age, the level of complaints before the operation and other illnesses.

It's good to realize that a knee replacement is an 'artificial joint'. You can expect a lot of improvement in most areas, but an arthroplasty can usually not match the 'normal' situation.

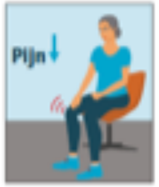

Pain

Almost everyone has less pain after the operation. Despite this improvement, you may still experience some pain in your knee, especially during strenuous or stressful activities. One year after the operation, 43% of patients experiences no pain at all.

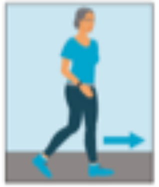

Walking

Walking short and medium distances improves a lot for most people. Long distances of a few kilometers often remain more difficult. Approximately 40% of people can walk as far as they want without being restricted by their knee.

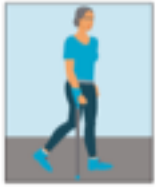

Walk with crutches or a stick

If you are walking with crutches or a stick before the operation because of the knee problems, chances are that this is not necessery anymore after the operation. A year after the operation, 1 in 20 people needs walking support.

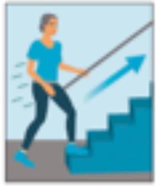

Climbing stairs

The ability to climb stairs usually improves after knee replacement, it hurts less and goes faster. About half of the people still need the banister. Climbing stairs remains slower than in people without total knee arthroplasty

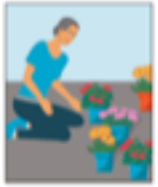

Kneeling and squatting

A total knee arthroplasty is not very suitable for kneeling and squatting. 1 out of 5 patients can kneel and squat after surgery without complaints. 1 out of 3 3 patients can not kneel or crouch at all after the operation.

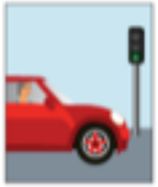

Use public transport or drive

Traveling by public transport or by car is generally without problems after knee replacement. You can usually drive yourself after 6 weeks. After 3 months, this succeeds for almost everyone.

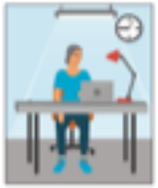

Work

Most people who work before their surgery, return to work after the operation. On average, you can return to work 8 to 12 weeks after the operation. Work that onvolves kneeling or crawling remains difficult. 1 to 2 out of 10 people no longer manages to work after the operation.

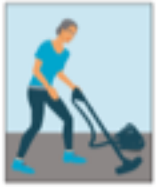

Activities of daily living

You can expect a lot of improvement for activities of daily living. 9 out of 10 people experience improvement in daily activities such as personal care and light housework. About 1 in 20 people remains to have problems in daily life.

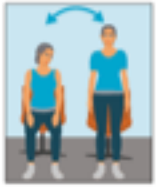

Change position

After the operation it is much easier to change position. Almost everyone does this faster after surgery and it's less painful. Almost everyone can stretch his or her leg completely after the operation.

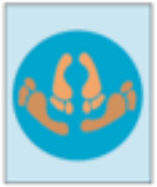

Sexual activity

In sexual activity a knee replacement rarely provides limitations. Fewer than 2 in 100 people indicate that sexual activity is not possible because of his or her knee replacement.
